# Serum Klotho Is Elevated in Patients with Acute Myocardial Infarction and Could Predict Poor In-Hospital Prognosis

**DOI:** 10.3390/jcdd11090292

**Published:** 2024-09-20

**Authors:** Yuanyuan Pei, Wenfeng Huang, Lingjie Cao, Fengtao Yang, Cheng Chi, Jihong Zhu

**Affiliations:** Department of Emergency, Peking University People’s Hospital, Beijing 100044, China; peiyuanyuan@pkuph.edu.cn (Y.P.); wenfenghuang0816@163.com (W.H.); cao0604@126.com (L.C.); fftfoo@163.com (F.Y.)

**Keywords:** serum Klotho, elevation, acute myocardial infarction, poor prognosis

## Abstract

Introduction: Klotho has emerged as a potential protective factor for cardiovascular diseases recently. Nevertheless, the levels of serum Klotho in acute coronary syndrome (ACS) have not been reported. Hence, we undertook a study to investigate the potential correlation between serum Klotho and ACS patients. Method: This observational cohort study was conducted at Peking University People’s Hospital between May 2016 and April 2020. Upon admission, we collected the patients’ clinical data and conducted ELISA tests to measure their serum Klotho levels. Result: A total of 349 patients were enrolled in this study, including 14 patients with UA and 335 patients with AMI. We observed that serum Klotho levels were obviously higher in the AMI group compared to the UA group (median 479.8 vs. 233.8 pg/mL, *p* = 0.035). In addition, serum Klotho levels were positively correlated with cardiac function and more pronounced in patients who died in the hospital (median 721.1 vs. 468.3 pg/mL, *p* < 0.001). A logistic regression analysis indicated that age ≥ 78 years old, HR ≥ 90 bpm, Killip classification ≥ 3 grade, and serum Klotho > 645.0 pg/mL were risk factors for poor prognosis. Conclusions: Serum Klotho is obviously increased in patients with AMI and with a positive correlation with cardiac function, and its elevation could serve as a predictor of poor prognosis in ACS patients.

## 1. Introduction

The Klotho gene family consists of α-Klotho, β-Klotho, and γ-Klotho. The primary type of Klotho protein is called α-Klotho, which was first identified in 1997 by Kuro-o et al. It is a 130 kDa single-pass transmembrane glycoprotein predominantly expressed in the renal distal convoluted tubules [1,2]. Kuro-o et al. [3] reported that Klotho hypomorphic mice exhibited a multi-organ failure syndrome, including a shortened lifespan, arteriosclerosis, cardiomyopathy, and more. During the synthesis of a Klotho protein, the extracellular domain can be selectively cleaved into a small-molecular-weight secreted protein that enters the bloodstream, exerting biological effects for multiple organs, including pro-autophagy, antioxidative anti-apoptosis, and antifibrosis activities [1,2].

Previous research has found a significant association between higher plasma Klotho levels and younger age, female sex, and smoking [4,5,6,7]. Recent studies have suggested a potential link between Klotho and cardiac and renal function [8,9,10]. Klotho deficiency has been observed in acute kidney injury (AKI) rodent models and patients with chronic kidney disease (CKD) [8,9,10]. Clinical studies have shown that Klotho expression is significantly reduced in dialysis patients. Additionally, lower Klotho levels are associated with long-term cardiovascular (CV) events, such as CV death and heart failure rehospitalization, suggesting a potential protective effect of Klotho on the heart [11,12].

In recent years, the role of Klotho in heart disease has garnered attention. It has been observed that Klotho can reduce apoptosis and improve myocardial remodeling in uremic cardiomyopathy. Furthermore, it can ameliorate myocardial ischemia/reperfusion injury by reducing inflammation [13,14]. However, the expression levels of Klotho in acute cardiovascular disease have not been reported. Therefore, we conducted a study to investigate the potential correlation between serum Klotho and outcomes in patients with acute coronary syndrome (ACS). 

## 2. Materials and Methods

### 2.1. Research Subjects

This prospective observational study was conducted at Peking University People’s Hospital, Beijing, China. It included a total of 349 consecutive patients admitted to the emergency department between May 2016 and April 2020, who were diagnosed with ACS. Exclusion criteria included CKD at stage 4 to 5, septic shock, and patients who died or were discharged within 48 h of admission. 

This study has received approval from the hospital ethics committee (No. 2022PHB061-001) and was implemented in accordance with the ethical guidelines for clinical research in China. Patients or their family members were provided with comprehensive information about this study and signed informed consent forms. STROBE-checklist, see Supplementary Materials.

### 2.2. Definition and Collection of Clinical Data

ACS includes unstable angina (UA) and acute myocardial infarction (AMI). UA was diagnosed according to a typical history of chest pain and diagnostic electrocardiographic changes according to the 2014 AHA/ACC Guideline of Non-ST-Elevation Acute Coronary Syndromes [15]. Meanwhile, the diagnosis of AMI should be accompanied by the presence of acute myocardial injury detected by abnormal cardiac biomarkers and the presence of acute myocardial ischemia [16]. The Killip classification was used to assess the cardiac function of patients with AMI. CKD was in accordance with the definition of the National Kidney Foundation, as kidney damage or GFR of less than 60 mL/min per 1.73 m^2^ for at least 3 months [17].

The patient records were carefully collected to gather comprehensive data on baseline characteristics, which included age, gender, co-morbidities, initial blood pressure and heart rate, Killip classification, cardiac complications, laboratory tests, the occurrence of emergent percutaneous coronary intervention (PCI) or coronary artery bypass graft (CABG) surgery, the need for intra-aortic balloon pump (IABP) treatment, temporary pacemaker implantation, and the use of mechanical ventilation and medications. The left ventricular ejection fraction (LVEF) was measured using the modified Simpson’s method with echocardiography within the first 24 h of hospitalization. The statistical analysis utilized the peak levels of troponin I (TNI) and creatine kinase-MB (CK-MB). The maximum daily doses of intravenous loop diuretics were all converted to furosemide, and the specific convertion method was as follows: 20 mg torsemide was approximately equal to 40 mg furosemide. All patients received standard antithrombotic therapy and cardioprotective drugs in accordance with current guidelines [15].

### 2.3. Serum Sampling and Measurement of Serum Klotho

Serum samples for Klotho were taken instantly in patients diagnosed with ACS in the emergency department. All samples were centrifuged at 1500 rpm for 10 min and then stored at −80 °C for further detection. All biomarkers were measured in duplicate using a single enzyme-linked immunosorbent assay (ELISA) according to the manufacturer’s instructions. A Klotho ELISA kit was obtained from R&D Systems (DY5334-05).

### 2.4. Statistical Analysis

The primary analysis compared the in-hospital death group with the discharge group. All descriptive statistics were summarized and described as the mean ± standard deviation or the median (25~75%). Continuous variables were compared by an independent sample t test or the Mann–Whitney U test. The categorical data were tested by the Chi-square test or Fisher’s exact test. The Spearman test was used to analyze the correlation between Klotho and renal function, where *p* < 0.05 was considered statistically significant.

A receiver operating characteristic (ROC) analysis was employed to investigate the predictive ability of Klotho in determining the prognosis of patients with ACS at admission. Discrimination was evaluated based on the area under the receiver operating characteristic curve (AUC). The AUCs were categorized as follows: 0.90~1.00 indicating excellent performance, 0.80~0.89 indicating good performance, 0.70~0.79 indicating fair performance, 0.60~0.69 indicating poor performance, and 0.50~0.59 indicating no useful performance [18]. Additionally, the AUC analysis was conducted to determine the optimal cut-off values, sensitivity, specificity, and cut-off points, which were calculated by identifying the best Youden index [18].

The independent predictors for poor prognosis were identified using logistic regression, and the significant factors in the univariate analysis combined with the clinical condition were included. Binary logistic regression models were generated using the Enter mode, and the association measures were calculated (adjusted odds ratio) with a confidence interval (CI) of 95%. *p* > 0.05 would indicate a good fit for the model. All analyses were performed with SPSS 25.0 software.

## 3. Results

### 3.1. Baseline Characteristics

Overall, 349 patients were included in this study. Among them, 14 patients had UA, while the remaining 335 patients had AMI. The in-hospital mortality was 3.4% (12/349). The demographic and baseline clinical characteristics of the patients are presented in Table 1. Compared to the discharge group, the in-hospital death group was obviously older, with massive myocardial infarctions, a higher Killip classification, more cardiac complications, worse renal function, the lower usage of an angiotensin-converting enzyme inhibitors/angiotensin receptor blocker (ACEI/ARB) and statins, and larger doses of diuretics. In addition, none of the patients who died in the hospital received PCI treatment.

### 3.2. The Levels of Serum Klotho Analyzed in Different Groups

As depicted in Table 2, an intergroup comparison based on gender and smoking history revealed a decrease in Klotho expression in male patients (median 458.8 vs. 518.1 pg/mL, *p* = 0.009) as well as in smokers (median 458.8 vs. 509.8 pg/mL, *p* = 0.011). Additionally, we divided ACS patients into two groups: the UA group and the AMI group. Interestingly, Klotho expression levels were significantly increased in the AMI group compared to the UA group (median 479.8 vs. 233.8 pg/mL, *p* = 0.035). Furthermore, a more pronounced elevation in Klotho levels was observed in patients who unfortunately passed away during their hospital stay (median 721.7 vs. 468.3 pg/mL, *p* < 0.001).

### 3.3. The Correlation between Serum Klotho and Cardiac–Renal Function

As displayed in Table 3, the levels of serum Klotho were more closely associated with cardiac function in ACS patients, showing a positive correlation with heart rate, Killip classification, and brain natriuretic peptide (BNP) levels. However, there was no statistical difference between Klotho and kidney-related markers, including blood urea nitrogen, serum creatinine, and eGFR.

### 3.4. Discrimination Performance of Serum Klotho for Prognosis in ACS Patients

**Figure 1:**
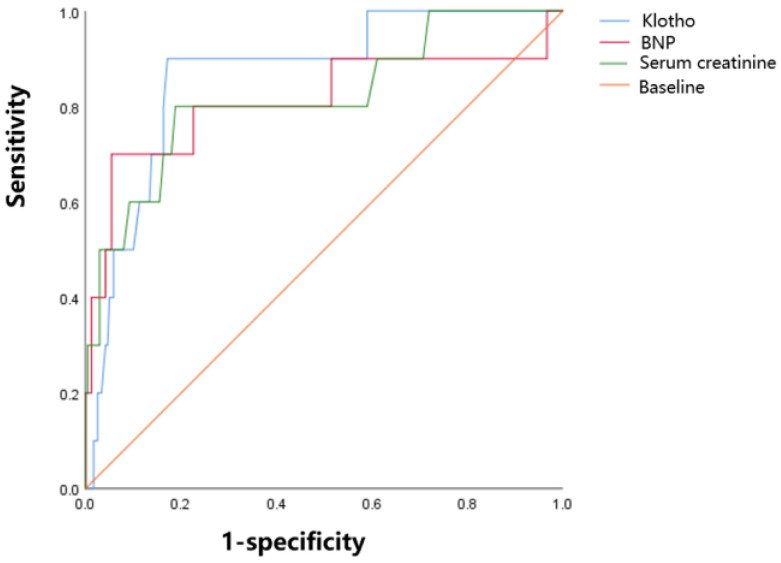
The figure on the left is the comparison of discrimination performance of Klotho, BNP, and SCr at admission regarding the in-hospital outcome. Klotho is blue, BNP is red, and SCr is green. The respective AUCs are 0.865 (*p* < 0.001), 0.811 (*p* = 0.001), and 0.819 (*p* =0.001).

As shown in Table 4 and Figure 1, when compared to serum creatinine and BNP, AUCs illustrated that serum Klotho possessed a modest discriminative ability in predicting the poor prognosis of ACS patients (AUC 0.865, 95% CI, 0.761–0.969, *p* < 0.001). The cut-off value of Klotho was 645.0 pg/mL, with a sensitivity of 0.900 and a specificity of 0.828 for in-hospital death.

### 3.5. Risk Factors of Poor Prognosis

We developed a regression model to elucidate the risk factors associated with poor prognosis during hospitalization. The final regression model included the following variables: age (≥78 years old) (OR = 8.169, 95% CI 1.199–55.672, *p* = 0.032), HR (≥90 bpm) (OR = 12.107, 95% CI 1.617–90.658, *p* = 0.015), Killip classification (≥3 grade) (OR = 16.590, 95% CI 3.037–90.608, *p* = 0.000), serum creatinine (≥93.5 μmol/L) (OR = 2.707, 95% CI 0.426–17.213, *p* = 0.291) and Klotho (>645.0 pg/mL) (OR = 6.017, 95% CI 1.108–35.555, *p* = 0.048) [Table 5].

## 4. Discussion

In this study, we attempted to investigate the serum levels of Klotho expression in patients diagnosed with ACS. Additionally, we conducted a preliminary analysis to determine its correlation with cardiac and renal function, as well as its impact on prognosis. The main results could be summarized as follows: 1. Serum Klotho was observed obviously elevated in patients with AMI when compared with patients with UA. 2. Levels of serum Klotho were more associated with cardiac function, showing a positive correlation with BNP levels but not related to renal function. 3. Klotho was pronounced increased in patients who died in hospital (median 721.1 vs. 468.3 pg/mL) and could predict poor prognosis.

Previous research has shown that lower levels of Klotho are linked to adverse cardiovascular (CV) events. In the PEACE trial conducted by Brian A Bergmark et al. [19], it was discovered that a low concentration of Klotho was strongly associated with an increased risk of CV death or hospitalization for heart failure in individuals with stable ischemic heart disease over a six-year follow-up period (adjusted hazard ratio 2.62) [19]. Additionally, the InCHIANTI study reported that higher plasma concentrations of Klotho were independently associated with a lower likelihood of having CV disease in a cross-sectional study involving 1023 adults living in the community [20]. Cai J. et al. [21] also found that a higher serum Klotho concentration was associated with a lower risk of heart failure. Furthermore, a study conducted in Spain indicated that higher plasma-soluble Klotho levels were associated with a lower cardiometabolic risk score in healthy, sedentary, middle-aged adults [22]. Moreover, basic research has observed that supplementing with Klotho could protect against indoxyl sulfate-mediated cardiac hypertrophy in mice and contribute to cardiac protection during ischemia/reperfusion injury in human cardiomyocytes [12,13]. Kai Chen et al. [23] revealed that Klotho deficiency induced cardiac aging by impairing the Nrf2-GR pathway, and supplements of Klotho prevented excessive oxidative stress, apoptosis, and heart failure. All of these findings suggest that elevated levels of Klotho may be beneficial for heart health.

The evolution of Klotho in the development of acute coronary syndrome (ACS) has not been previously reported. In this study, we observed that serum Klotho is significantly higher in patients with AMI compared to those with UA, and this elevation is strongly associated with a poor prognosis. In terms of predicting in-hospital death, the discriminative ability of Klotho, as measured by AUC, is moderately strong (AUC 0.865, 95% CI, 0.761–0.969, *p* = 0.000), surpassing that of serum creatinine and BNP. The cut-off value of Klotho is 645.0 pg/mL, and its sensitivity and specificity for in-hospital death are 0.900 and 0.828. The logistic regression model indicates that ACS patients with Klotho levels greater than 645.0 pg/mL at admission have a 2.442-fold increased risk of in-hospital death. In addition, the poor prognosis group is obviously older but exhibits higher serum Klotho levels. Klotho is renowned for its anti-aging properties and is typically elevated in young individuals [1,2]. Therefore, the elevation of Klotho in older patients with poor prognosis is prone to be associated with AMI. Furthermore, consistent with previous studies, Klotho levels are significantly elevated in women [24].

There was a study conducted on the dynamic levels of Klotho in patients with acute heart failure (AHF). It disclosed that Klotho increased remarkably in AHF patients, and those admitted with higher levels of Klotho responded better to treatment. Interestingly, patients who showed improvement had a significant decrease in Klotho levels [23]. Consequently, the authors speculated that Klotho could potentially serve as a novel biomarker for assessing therapy responsiveness in AHF patients. Another study analyzed the association of serum FGF23 and Klotho in the progression of 287 patients with chronic heart failure (CHF) and cardiomyopathy (CMP) as the etiology. And Klotho expression was detected in 10 hearts of CMP patients compared with 10 healthy controls [24]. The results indicated that serum Klotho was not related to disease severity or progression in CHF. However, Klotho was expressed and upregulated in diseased hearts by Western blotting, which suggests the presence of local paracrine effects in the hearts of CMP patients [25]. In line with our research, these studies all observed the elevation phenomenon of Klotho expression in heart disease. They revealed that serum Klotho might be increased when acute cardiovascular events develop, and in patients with chronic heart disease, Klotho could be elevated in the heart through paracrine effects, rather than circulation.

Based on the above findings, we concluded that there is a possibility for Klotho to be produced as a compensatory response in order to protect the heart during ACS, assuming that Klotho acts as a protective factor. Klotho has been proven to have significant anti-inflammatory effects in previous studies [26,27]. Qi Mao et al. have reported the decreased concentrations in serum Klotho, which was negatively correlated with inflammatory cytokine IL-6 and IL-8 and related to the severity of coronary atherosclerosis disease in middle-aged and elderly patients [28]. A mouse model of cardiac–renal syndrome after renal ischemia/reperfusion showed that Klotho might alleviate cardiac outcomes by systematically preventing inflammation and increasing FGF23 [29]. In an observational study of patients diagnosed recurrent AMI with non-obstructed coronary artery (MINOCA), Ciliberti et al. [30] have demonstrated that these patients tend to have a more severe atherosclerosis progression, which has been shown to be associated with inflammation, and Klotho may have a critical role in reducing atherosclerotic plaque destabilization by inhibiting an activated inflammatory response, improving prognosis among high-risk cardiovascular populations eventually. Nevertheless, we are left wondering why patients with higher levels of Klotho exhibit a higher mortality rate in this study. It is worth noting that the death group tended to be older, had worse cardiac function, and more severe cardiac dysfunction. Further investigation is required to determine whether Klotho is a negative feedback regulator for the body’s self-protection.

This study has its limitations. First, the sample size of enrolled patients was relatively small, and all were from a single center. Second, Klotho was only detected on admission at emergency. With the vast majority of patients admitted to the cardiology department, we did not monitor the dynamic changes in Klotho. Third, the in-hospital deaths of ACS patients decreased significantly due to vascular revascularization interventions, which can lead to statistical bias.

## 5. Conclusions

In summary, this study provides the view that the Klotho expression could be compensatory elevated in AMI patients, and the overexpression is associated with poor nosocomial prognosis. Whether the increase in Klotho is a self-protective factor after AMI remains to be further studied.

## Figures and Tables

**Table 1:** Baseline characteristics in ACS patients.

Variables	Total (*n* = 349)	In-Hospital Death Group (*n* = 12)	Discharge Group (*n* = 337)	*p* Value
**Demography**				
Age (years)	64 (54, 75)	80 (75, 86)	64 (54, 74)	<0.001
Male (%)	265 (75.7)	9 (75.0)	256 (75.7)	0.590
Hypertension (%)	211 (60.3)	10 (83.3)	201 (59.5)	0.135
Diabetes mellitus (%)	105 (30.0)	2 (16.7)	103 (30.5)	0.522
Prior MI (%)	50 (14.3)	3 (25.0)	47 (13.9)	0.390
CHF (%)	4 (1.1)	1 (8.3)	3 (0.9)	0.131
CKD (%)	34 (9.7)	3 (25.0)	31 (9.2)	0.100
Cerebral infarction (%)	47 (13.4)	4 (33.3)	43 (12.7)	0.063
Previous PCI (%)	46 (13.1)	1 (8.3)	45 (13.3)	0.616
Previous CABG (%)	8 (2.3)	1 (8.3)	7 (2.1)	0.246
Chronic lung diseases (%)	10 (2.9)	1 (8.3)	9 (2.7)	0.298
Tumor (%)	19 (5.4)	2 (16.7)	17 (5.0)	0.133
Dislipidemia (%)	75 (21.4)	0 (0.0)	75 (22.2)	0.077
**Clinical presentation**				
STEMI (%)	209 (59.7)	6 (50.0)	203 (60.1)	0.555
Extensive anterior myocardial infarction (%)	55 (15.7)	5 (41.7)	50 (14.8)	0.026
Killip classification ≥ III stage (%)	27 (7.7)	9 (75.0)	18 (5.3)	<0.001
Heart rate (bpm)	76 (68, 89)	110 (95, 124)	76 (68, 87)	<0.001
Systolic pressure (mmHg)	124 ± 27	124 ± 44	124 ± 27	0.975
Diastolic pressure (mmHg)	72 ± 16	72 ± 27	71 ± 15	0.945
Cardiogenic shock (%)	15 (4.3)	4 (33.3)	11 (3.3)	0.001
Ventricular tachycardia (%)	20 (5.7)	0 (0.0)	20 (5.9)	0.385
Ventricular fibrillation (%)	17 (4.9)	4 (33.3)	13 (3.8)	0.002
Atrial fibrillation (%)	27 (7.7)	3 (25.0)	24 (7.1)	0.056
Atrioventricular block (%)	10 (2.9)	0 (0.0)	10 (3.0)	0.545
Smokers (%)	214 (61.3)	4 (33.3)	210 (62.7)	0.042
Contrast volume (mL)	200 (100, 200)	0 (0, 100)	200 (100, 205)	0.003
**Laboratory tests**				
Hemoglobin (g/L)	133 (118, 144)	116 (105, 133)	133 (119, 144)	0.058
WBC (×10^9^/L)	8.9 (6.9, 11.5)	12.3 (9.1, 16.1)	8.8 (6.9, 11.4)	0.027
PLT (×10^9^/L)	197 (162, 243)	195 (116, 252)	197 (163, 243)	0.484
SCr in admission (μmol/L)	76.0 (63.0, 90.0)	103.5 (84.0, 172.0)	75.0 (62.5, 88.0)	<0.001
eGFR (mL/min × 1.73 m^2^)	88.54 (68.62, 100.07)	41.87 (26.38, 68.62)	89.35 (70.24, 100.81)	<0.001
BUN (mmol/l)	5.72 (4.42, 7.38)	10.74 (8.10, 15.35)	5.67 (4.38, 7.06)	<0.001
TNI (ng/mL)	11.1 (2.47, 47.37)	15.2 (1.5, 25.1)	10.9 (2.5, 49.4)	0.700
CK-MB (U/L)	42.4 (8.1, 188.7)	25.1 (12.4, 80.0)	43.3 (8.0, 190.6)	0.358
BNP (pg/mL)	195 (82, 409)	1129 (350, 2917)	190 (81, 360)	0.001
FBG (mmol/L)	6.50 (5.32, 8.54)	10.2 (7.3, 12.3)	6.4 (5.3, 8.4)	0.012
Albumin (g/L)	38.0 (35.0, 40.9)	32.9 (28.4, 36.5)	38.1 (35.0, 41.0)	0.002
CHO (mmol/L)	4.30 ± 1.14	3.65 ± 0.63	4.32 ± 1.14	0.053
TG (mmol/L)	1.48 (1.08, 2.17)	1.17 (0.76, 1.45)	1.49 (1.08, 2.20)	0.051
LDL (mmol/L)	2.69 ± 0.94	2.03 ± 0.60	2.71 ± 0.94	0.017
HDL (mmol/L)	0.97 (0.82, 1.15)	0.95 (0.68, 1.08)	0.97 (0.83, 1.15)	0.198
LVEF (%)	62 (56, 68)	53 (40, 65)	63 (56, 68)	0.030
**Therapies**				
Use of furosemide (mg/d)	0 (0, 20)	140 (40, 285)	0 (0, 20)	<0.001
Intravenous isosorbide dinitrate (%)	229 (65.4)	9 (75.0)	220 (65.1)	0.555
Non-use of ACEI/ARB (%)	159 (45.4)	11 (91.7)	148 (43.8)	0.001
Non-use of β-blockers (%)	79 (22.6)	4 (33.3)	75 (22.2)	0.479
Non-use of statins (%)	21 (6.0)	4 (33.3)	17 (5.0)	0.003
Vasoactive medication (%)	56 (16.0)	6 (50.0)	50 (14.8)	0.006
IABP (%)	16 (4.6)	1 (8.3)	15 (4.4)	0.435
In-hospital PCI (%)	220 (62.9)	0 (0.0)	220 (65.0)	0.000
In-hospital CABG (%)	24 (6.9)	1 (8.3)	23 (6.8)	0.580
In-hospital temporary pacemaker (%)	1 (0.3)	0 (0.0)	1 (0.3)	0.966
In-hospital mechanical ventilation (%)	21 (6.0)	3 (25.0)	18 (5.3)	0.029
Thrombolysis therapy (%)	5 (1.4)	0 (0.0)	5 (1.5)	0.671
**Biomarker**				
Klotho (pg/mL)	477.8 (89.6, 589.8)	721.7 (597.0, 1007.1)	470.7 (85.8, 582.4)	<0.001

Data expressed as mean ± standard deviation, n (%), or median (interquartile range). MI, myocardial infarction. CHF, congestive heart failure. CKD, chronic kidney disease. PCI, percutaneous coronary intervention. CABG, coronary artery bypass grafting. STEMI, ST-segment elevation myocardial infarction. WBC, white blood cell. PLT, platelet. SCr, serum creatinine. BUN, blood urea nitrogen. eGFR, estimated glomerular filtration rate. TNI, troponin I. BNP, brain natriuretic peptide. CK-MB, creatine kinase-MB. FBG, fasting blood glucose. LVEF, left ventricular ejection fraction. CHO, cholesterol. TG, triglyceride. LDL, low-density lipoprotein. HDL, high-density lipoprotein. ACEI/ARB, angiotensin-converting enzyme inhibitors/angiotensin receptor blocker. The above therapies are all implemented during hospitalization. Compared with the in-hospital death group, *p* < 0.05 was considered significant.

**Table 2:** Serum Klotho levels in different groups.

Klotho (pg/mL)	Smokers	Non-smokers	*p* value
	458.8 (75.0, 561.1)	509.8 (182.3, 640.0)	0.011
	Male	Female	0.009
	458.8 (78.1, 575.4)	518.1 (277.2, 644.9)	
	AMI	UA	0.035
	479.8 (93.4, 590.9)	233.8 (61.6, 477.8)	
	Discharge group	In-hospital death group	<0.001
	468.3 (85.8, 582.4)	721.7 (567.0, 1007.1)	

AMI, acute myocardial infarction. UA, unstable angina. *p* < 0.05 was considered significant.

**Table 3:** The correlation between Klotho and cardiac–renal function.

	Klotho	
	r	*p* Value
Heart rate	0.111	0.039
Killip classification	0.191	0.000
TNI	0.053	0.323
BNP	0.237	0.000
LVEF	0.001	0.979
BUN	0.015	0.785
SCr	0.069	0.197
eGFR	−0.102	0.056

TNI, troponin I. BNP, brain natriuretic peptide. LVEF, left ventricular ejection fraction. Cr, serum creatinine. BUN, blood urea nitrogen. eGFR, estimated glomerular filtration rate. *p* < 0.05 was considered significant.

**Table 4:** The AUCs of serum Klotho, BNP and SCr for predicting in-hospital mortality.

	AUC	95% CI	*p* Value	Cut-Off Value	Sensitivity	Specificity
SCr (μmol/L)	0.819	0.664–0.975	0.001	94.5	0.800	0.812
BNP (pg/mL)	0.812	0.624–0.999	0.001	967	0.700	0.946
Klotho (pg/mL)	0.865	0.761–0.969	0.000	645.0	0.900	0.828

AUC, area under the receiver operating characteristic curves; CI, confidence interval.

**Table 5:** The logistic regression model for predicting poor prognosis.

Correlates	OR	95% CI	*p* Value
Age (≥78 years old)	8.169	1.199–55.672	0.032
Heart rate (≥90 bpm)	12.107	1.617–90.658	0.015
Killip classification (≥ 3 grade)	16.590	3.037–90.608	0.000
Serum creatinine (≥93.5 μmol/L)	2.707	0.426–17.213	0.291
Klotho (>645.0 pg/mL)	6.017	1.108–35.555	0.048

## Data Availability

The datasets used or analyzed for this study are available from the corresponding author upon reasonable request.

## Supplementary Materials

The following supporting information can be downloaded at: https://www.mdpi.com/article/10.3390/jcdd11090292/s1.
