# Supplementary material for: Serum Klotho Is Elevated in Patients with Acute Myocardial Infarction and Could Predict Poor In-Hospital Prognosis
Source: J Cardiovasc Dev Dis. 2024 Sep 20;11(9):292. doi: 10.3390/jcdd11090292 (PMC11432139; doi:10.3390/jcdd11090292)
Supplement: Supplementary file 1 [file jcdd-11-00292-s001.zip › jcdd-3123634-supplementary.pdf]

STROBE Statement—checklist of items that should be included in reports of observational studies

	Item No	Recommendation
**Title and abstract**	1	(*a*) Indicate the study's design with a commonly used term in the title or the abstract<br>Serum Klotho Is Elevated in Patients with Acute Myocardial Infarction and Could Predict Poor In-Hospital Prognosis
		(*b*) Provide in the abstract an informative and balanced summary of what was done and what was found<br>This observational cohort study was conducted at Peking University People's Hospital between May 2016 and April 2020. Upon admission, we collected the patients' clinical data and conducted ELISA tests to measure their serum Klotho levels. Serum Klotho is obviously increased in patients with AMI and with a positive correlation with cardiac function, and its elevation could serve as a predictor of poor prognosis in patients with acute coronary syndrome(ACS).
**Introduction**		
Background/rationale	2	Explain the scientific background and rationale for the investigation being reported<br>Recent studies have suggested a potential link between Klotho and cardiac function. Lower Klotho levels are associated with long-term cardiovascular (CV) events, such as CV death and heart failure rehospitalization, suggesting a potential protective effect of Klotho on the heart. In recent years, the role of Klotho in heart disease has garnered attention. It has been observed that Klotho can reduce apoptosis and improve myocardial remodeling in uremic cardiomyopathy. Furthermore, it can ameliorate myocardial ischemia/reperfusion injury by reducing inflammation. However, the expression levels of Klotho in acute cardiovascular disease have not been reported.
Objectives	3	State specific objectives, including any prespecified hypotheses<br>In this study, we conducted a study to investigate the potential correlation between serum Klotho and outcomes in patients with ACS.
**Methods**		
Study design	4	Present key elements of study design early in the paper<br>This observational cohort study was conducted at Peking University People's Hospital between May 2016 and April 2020. Clinical data was collected and serum Klotho levels were measured BY ELISA tests. All analyses were performed with SPSS 25.0 software.
Setting	5	Describe the setting, locations, and relevant dates, including periods of recruitment, exposure, follow-up, and data collection<br>This prospective observational study was conducted at Peking University People's Hospital, Beijing, China. It included a total of 349 consecutive patients admitted to the emergency department between May 2016 and April 2020, who were diagnosed with ACS.
Participants	6	(*a*) Cohort study—Give the eligibility criteria, and the sources and methods of selection of participants. Describe methods of follow-up<br>Case-control study—Give the eligibility criteria, and the sources and methods of case ascertainment and control selection. Give the rationale for the choice of cases and controls<br>Cross-sectional study—Give the eligibility criteria, and the sources and methods of selection of participants

		Patients diagnosed with ACS were enrolled. Exclusion criteria included CKD at stage 4 to 5, septic shock, and patients who died or were discharged within 48 h of admission.
		(*b*) *Cohort study*—For matched studies, give matching criteria and number of exposed and unexposed<br>*Case-control study*—For matched studies, give matching criteria and the number of controls per case
Variables	7	Clearly define all outcomes, exposures, predictors, potential confounders, and effect modifiers. Give diagnostic criteria, if applicable<br>ACS includes unstable angina (UA) and acute myocardial infarction (AMI). UA was diagnosed according to a typical history of chest pain and diagnostic electrocar-diographic changes according to the 2014 AHA/ACC Guideline of Non-ST-Elevation Acute Coronary Syndromes. And In-hospital outcomes were recorded.
Data sources/ measurement	8*	For each variable of interest, give sources of data and details of methods of assessment (measurement). Describe comparability of assessment methods if there is more than one group<br>The patient records were carefully collected to gather comprehensive data on baseline characteristics, which included age, gender, co-morbidities, initial blood pressure and heart rate, Killip classification, cardiac complications, laboratory tests, the occurrence of emergent percutaneous coronary intervention (PCI) or coronary artery bypass graft (CABG) surgery, the need for intra-aortic balloon pump (IABP) treatment, temporary pacemaker implantation, and the use of mechanical ventilation and medications. In-hospital outcomes were recorded. Serum klotho was tested by ELISA. The primary analysis compared the in-hospital death group with the discharge group.
Bias	9	Describe any efforts to address potential sources of bias
Study size	10	Explain how the study size was arrived at
Quantitative variables	11	Explain how quantitative variables were handled in the analyses. If applicable, describe which groupings were chosen and why
Statistical methods	12	(*a*) Describe all statistical methods, including those used to control for confounding<br>All descriptive statistics were summarized and described as the mean ± standard devi-ation or the median (25~75%). Continuous variables were compared by an independent sample t test or the Mann–Whitney U test. The categorical data were tested by the Chi-square test or Fisher’s exact test. The Spearman test was used to analyze the correlation between Klotho and renal function, where $p < 0.05$ was considered statistically significant. The independent predictors for poor prognosis were identified using logistic regression, and the significant factors in the univariate analysis combined with the clin-ical condition were included. Binary logistic regression models were generated using the Enter mode, and the association measures were calculated (adjusted odds ratio) with a confidence interval (CI) of 95%. $p > 0.05$ would indicate a good fit for the model. All analyses were performed with SPSS 25.0 software.
		(*b*) Describe any methods used to examine subgroups and interactions
		(*c*) Explain how missing data were addressed
		(*d*) *Cohort study—If applicable, explain how loss to follow-up was addressed*<br>*Case-control study—If applicable, explain how matching of cases and controls was addressed*<br>*Cross-sectional study—If applicable, describe analytical methods taking account of sampling strategy*

		(*e*) Describe any sensitivity analyses<br>A receiver operating characteristic (ROC) analysis was employed to investigate the predictive ability of Klotho in determining the prognosis of patients with ACS at ad-mission. Discrimination was evaluated based on the area under the receiver operating characteristic curve (AUC). Additionally, the AUC analysis was conducted to determine the optimal cut-off values, sensitivity, specificity, and cut-off points, which were calculated by identifying the best Youden index

Continued on next page

**Results**		
Participants	13*	(a) Report numbers of individuals at each stage of study—eg numbers potentially eligible, examined for eligibility, confirmed eligible, included in the study, completing follow-up, and analysed<br>Overall, 349 patients were included in this study. Among them, 14 patients had UA, while the remaining 335 patients had AMI.
		(b) Give reasons for non-participation at each stage
		(c) Consider use of a flow diagram
Descriptive data	14*	(a) Give characteristics of study participants (eg demographic, clinical, social) and information on exposures and potential confounders<br>Compared to the discharge group, the in-hospital death group was obviously older, with massive myocardial infarctions, a higher Killip classification, more cardiac com-plications, worse renal function, the lower usage of an angiotensin-converting enzyme inhibitors/angiotensin receptor blocker (ACEI/ARB) and statins, and larger doses of diuretics. In addition, none of the patients who died in the hospital received PCI treatment.
		(b) Indicate number of participants with missing data for each variable of interest
		(c) *Cohort study*—Summarise follow-up time (eg, average and total amount)
Outcome data	15*	*Cohort study*—Report numbers of outcome events or summary measures over time
		Case-control study—Report numbers in each exposure category, or summary measures of exposure
		Cross-sectional study—Report numbers of outcome events or summary measures<br>*The in-hospital mortality was 3.4% (12/349).*
Main results	16	(*a*) Give unadjusted estimates and, if applicable, confounder-adjusted estimates and their precision (eg, 95% confidence interval). Make clear which confounders were adjusted for and why they were included<br>Risk factors related with in-hospital mortality included the following variables: age (⩾78 years old) (OR = 8.169, 95% CI 1.199–55.672, p = 0.032), HR (⩾90 bpm) (OR = 12.107, 95% CI 1.617–90.658, p = 0.015), Killip classification (⩾3 grade) (OR = 16.590, 95% CI 3.037–90.608, p = 0.000), serum creatinine (⩾93.5 μmol/L) (OR = 2.707, 95% CI 0.426–17.213, p = 0.291) and Klotho (>645.0 pg/mL) (OR = 6.017, 95% CI 1.108–35.555, p = 0.048)
		(*b*) Report category boundaries when continuous variables were categorized
		(*c*) If relevant, consider translating estimates of relative risk into absolute risk for a meaningful time period
Other analyses	17	Report other analyses done—eg analyses of subgroups and interactions, and sensitivity analyses
**Discussion**		
Key results	18	Summarise key results with reference to study objectives<br>1). Serum Klotho was observed obviously elevated in patients with AMI when com-pared with patients with UA. 2). Levels of serum Klotho were more associated with cardiac function,

		showing a positive correlation with BNP levels but not related to renal function. 3). Klotho was pronounced increased in patients who died in hospital (median 721.1 vs. 468.3 pg/mL) and could predict poor prognosis.
	19	Discuss limitations of the study, taking into account sources of potential bias or imprecision. Discuss both direction and magnitude of any potential bias<br>First, the sample size of enrolled patients was relatively small, and all were from a single center. Second, Klotho was only detected on admission at emergency. With the vast majority of patients admitted to the cardiology department, we did not monitor the dynamic changes in Klotho. Third, the in-hospital deaths of ACS patients decreased significantly due to vascular revascularization interventions, which can lead to statis-tical bias.
Interpretation	20	Give a cautious overall interpretation of results considering objectives, limitations, multiplicity of analyses, results from similar studies, and other relevant evidence<br>This study provides the view that the Klotho expression could be compensatory ele-vated in AMI patients, and the overexpression is associated with poor nosocomial prognosis. Whether the increase in Klotho is a self-protective factor after AMI remains to be further studied.
Generalisability	21	Discuss the generalisability (external validity) of the study results<br>More studies are needed to demonstrate the results.
**Other information**		
Funding	22	Give the source of funding and the role of the funders for the present study and, if applicable, for the original study on which the present article is based<br>This study was supported by Beijing Health Technology Achievements and Appropriate Technology Promotion Project (no. BHTPP2024029), research and development fund of Peking University Peo-ple's Hospital in 2022, Project No. RDJ 2022-18; Capital Characteristic Clinic Project in 2016, Project No. Z161100000516045; and the research and development fund of Peking University People's Hospital in 2021, Project No. PTU 2021-02.

*Give information separately for cases and controls in case-control studies and, if applicable, for exposed and unexposed groups in cohort and cross-sectional studies.

Note: An Explanation and Elaboration article discusses each checklist item and gives methodological background and published examples of transparent reporting. The STROBE checklist is best used in conjunction with this article (freely available on the Web sites of PLoS Medicine at http://www.plosmedicine.org/, Annals of Internal Medicine at http://www.annals.org/, and Epidemiology at http://www.epidem.com/). Information on the STROBE Initiative is available at www.strobe-statement.org.
